# Trilled /r/ is associated with roughness, linking sound and touch across spoken languages

**DOI:** 10.1038/s41598-021-04311-7

**Published:** 2022-01-20

**Authors:** Bodo Winter, Márton Sóskuthy, Marcus Perlman, Mark Dingemanse

**Affiliations:** 1 Department of English Language and Linguistics, University of Birmingham, Birmingham, UK; 2 Department of Linguistics, University of British Columbia, Vancouver, Canada; 3 Centre for Language Studies, Radboud University, Nijmegen, The Netherlands

**Keywords:** Psychology, Human behaviour

## Abstract

Cross-modal integration between sound and texture is important to perception and action. Here we show this has repercussions for the structure of spoken languages. We present a new statistical universal linking speech with the evolutionarily ancient sense of touch. Words that express roughness—the primary perceptual dimension of texture—are highly likely to feature a trilled /r/, the most commonly occurring rhotic consonant. In four studies, we show the pattern to be extremely robust, being the first widespread pattern of iconicity documented not just across a large, diverse sample of the world’s spoken languages, but also across numerous sensory words within languages. Our deep analysis of Indo-European languages and Proto-Indo-European roots indicates remarkable historical stability of the pattern, which appears to date back at least 6000 years.

## Introduction

Iconicity, the perceived resemblance between aspects of the form of a signal and aspects of its meaning, is now widely recognized to be a core property of all human languages, spoken and signed^1,2^. When an iconic form-meaning mapping is rooted in a perceptual analogy that is shared across people from different language backgrounds, universal tendencies in the phonological structure of words can emerge^3–5^. One perceptual modality that has so far received relatively little attention in cross-linguistic studies of iconicity is touch, even though this modality is evolutionary ancient and of critical biological and cultural importance^6–8^. For humans, the texture of objects and materials is an important characteristic that determines how they are used and evaluated^9,10^. Not surprisingly then, languages generally have dedicated subsets of their vocabulary for the description of texture^11,12^, potentially consisting of several hundred words as in the case of English^13^, e.g., *rough, smooth, hard, soft, coarse, cottony, fuzzy, silky, oily*, and more. Here, we focus on the textural dimension of roughness, which persistently emerges as the dominant perceptual dimension of surface touch when different textures are compared to each other^14,15^. We investigate whether the texture vocabulary for roughness is characterized by iconicity.

Although sound-meaning correspondences in texture vocabulary have not been widely studied, there are reasons to expect such associations, specifically with respect to roughness. There is extensive evidence from both behavioral^16–21^ and neural^22–24^ studies demonstrating that touch is highly integrated with audition. Audiotactile interactions have also been shown for roughness perception^19^, and people can reliably judge textural roughness from acoustic cues alone^25^. Moreover, people readily classify sounds in terms of ‘roughness’^26–28^. In English, the words *rough* and *smooth* are two very common descriptors of auditory qualities^29^, and, more generally, touch words are amongst the most frequent adjectives used to describe sound^30–32^. Given these extensive perceptual and linguistic interactions between sound and touch, there is clear potential for spoken words to directly mimic aspects of surface perception. Indeed, experimental research across spoken languages has found that speakers reliably associate particular speech sounds with particular texture concepts^33–35^, and English words with touch-related meanings (e.g., *mushy*, *crisp*) are rated to be amongst the most iconic words compared to other sensory words^36,37^. Finally, touch has been shown to emerge as an important semantic dimension when participants perform semantic grouping tasks on highly iconic words^38^.

Here, we report a striking cross-linguistic link between words denoting roughness and the occurrence of rhotic consonants (variants of /r/), a phoneme class that is found in about three-quarters of the world’s spoken languages^39^. We find that trilled /r/, the most commonly occurring rhotic^40^, is overwhelmingly more likely to occur in words for rough surfaces than smooth surfaces. In four studies, we show evidence for this pattern across a large set of sensory words in English (Study 1), in Hungarian (Study 2), in a genetically diverse sample of 331 spoken languages from 84 families (Study 3), and in 38 modern Indo-European languages as well as in reconstructed Proto-Indo-European roots (Study 4). Our data show that the trilled /r/-roughness pattern is prevalent across the world’s spoken languages (breadth) and across lots of words within a language (depth). Our comparison of Proto-Indo-European with modern Indo-European languages shows that the pattern also remains remarkably stable over historical time.

## Results

### English adjectives

In Study 1, we examined 99 English adjectives rated in prior work for roughness^13^. The set of words is a subset of 440 descriptors that were generated by asking American English speakers to “list as many adjectives as possible that described an object through the sense of touch”. These words were then rated for their relevance to the perceptual dimensions of roughness, hardness, motion, temperature, weight, size, and shape. The set considered here is the set of words for which roughness is relevant, which was the largest set of descriptors (e.g., the set of primarily temperature-related descriptors was smaller). The roughness-related words were subsequently rated on a scale from − 7 (smooth) to + 7 (rough). The ten roughest words in this dataset include *abrasive, barbed, jagged, rough, spiky, thorny*, *harsh, coarse, prickly, scratchy*; the ten smoothest words include *smooth, lubricated, oily, slippery, silky, slick, polished, satiny, velvety,* and *fine*.

**Figure 1:**
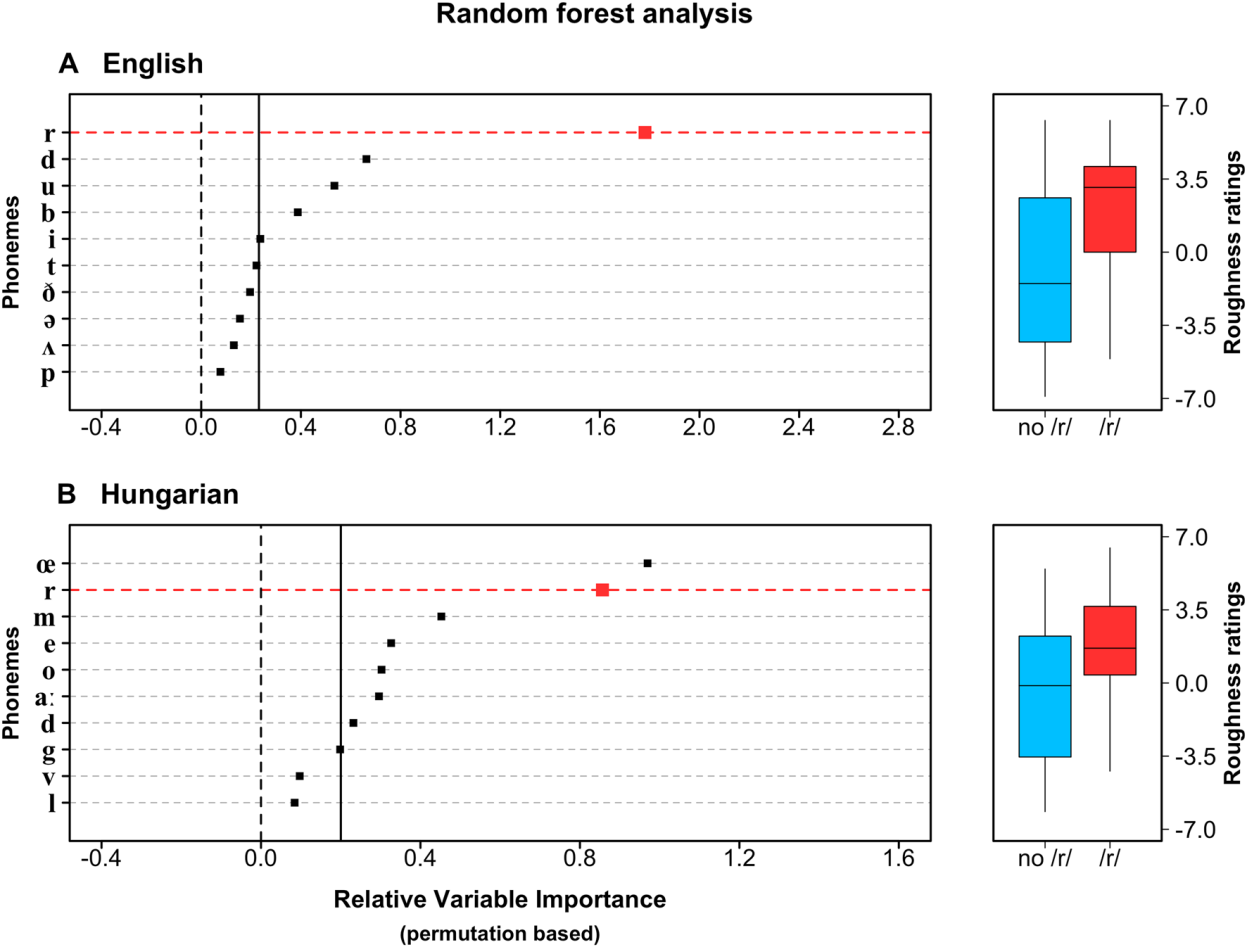
How phonemes relate to roughness ratings. Left: The ten most predictive phonemes in a random forest analysis for (**a**) English and (**b**) Hungarian; the vertical black line corresponds to the absolute value of the least predictive phoneme, which is a heuristic cut-off rule for predictors that do not contribute^42^. Right: Boxplots (whiskers = smallest, largest value within 1.5 × IQR) for words with and without /r/ in (**a**) English and (**b**) Hungarian.

We first assessed whether the presence/absence of a particular contrastive sound (phoneme) of English (e.g., /r/, /l/, /i/, etc.) is predictive of a word’s roughness rating. We used random forests^41^, a statistical method that performs well with small data sets when there are many potentially collinear predictor variables^42^. This analysis showed the presence/absence of the phoneme /r/ to be the most strongly predictive of semantic roughness/smoothness (Fig. 1a). The association between /r/ and roughness is also clear when we discretize the roughness continuum, comparing the incidence of /r/ in rough words (roughness rating ≥ 0) versus smooth words (roughness rating < 0). A Bayesian logistic regression indicates that 63% of rough words contain an /r/, with a 95% credible interval of [51%, 75%]. In contrast, only 30% [17%, 44%] of smooth words contain an /r/. The estimated difference in the incidence of /r/ between the two groups of words is 33% [15%, 51%] (99.95% of the posterior samples over zero). On average, words with /r/ are rated 2.75 higher on the roughness scale than words without /r/, which is a large effect size (Cohen’s *d* = 0.76).

A further indicator of the strong association between /r/ and roughness is the fact that the estimated relative frequency of /r/ in 58 rough adjectives (63%) is unusually high when compared to a baseline of 5,806 English adjectives (35%), as well as when compared to a baseline of 386 sensory adjectives (32%)^43^. This shows that it is specifically rough words for which /r/ is over-represented.

### Hungarian adjectives

If this pattern in English vocabulary derives from perceptual correspondence between /r/ and roughness (i.e. iconicity), and is not simply a historical idiosyncrasy, then we would expect to find a similar pattern in unrelated spoken languages. Therefore, in Study 2, we replicated the above analysis for Hungarian, a language of the Finno-Ugric family. We collected novel roughness ratings for 85 Hungarian sensory adjectives, including only words that are not borrowed from Indo-European. Similar to English, the presence of /r/ was found to be a strong predictor of roughness in a random forest analysis (Fig. 1b). While /œ/ is ranked above /r/ in terms of predictive strength, this phoneme only occurs in three words, as opposed to 29 words for /r/. Splitting the roughness scale into two categories as we did for English, the /r/ sound occurs in 57% [42%, 71%] of rough words but only 22% [9%, 40%] of smooth words, with an estimated difference of 35% [12%, 55%] (99.8% of the posterior samples over zero). On average, Hungarian words with /r/ have a roughness rating that is 2.27 higher than words without /r/ (Cohen’s d = 0.78).

Importantly, however, the /r/ phoneme has distinct phonetic realizations in English and Hungarian. ‘Rhotics’, the diverse class of sounds denoted by /r/, involve different manners of production, often showing considerable variation even within a single language^44^. In Hungarian, /r/ is an apical trill, also known as a “rolled r”^45,46^, a sound that is produced with aerodynamically controlled vibrations of the tongue which create rapid periodic interruptions of air flow^47,48^. In contrast, in most varieties of present-day English, the /r/ phoneme is a rhotic approximant. In this un-trilled form, the articulators approach each other, but without creating a full closure. Despite the different realization in these two languages, we considered that trilling may still be the key phonetic feature underlying both results. This is for two reasons: first, auditory roughness is characterized by amplitude variations^27^ that are also characteristic of trills, which, due to their intermittent airflow, vibrate with a frequency of 20–30 Hz depending on the language^49,50^. There is a clear analogy between textural roughness, which is characterized by the spatial frequency of grating patterns^51–53^ and trills, which are the only speech sounds characterized by intermittency. Second, there is evidence that the phonetic realization in earlier stages of the English language was an apical trill as well, a realization that survived as a positional variant well into the eighteen–nineteenth centuries^54,55^. Thus, while the un-trilled rhotic approximant of modern-day English may no longer bear an iconic link to roughness, the association in the lexicon may be left over from a past stage of the language.

### Large-scale cross-linguistic comparison

**Figure 2:**
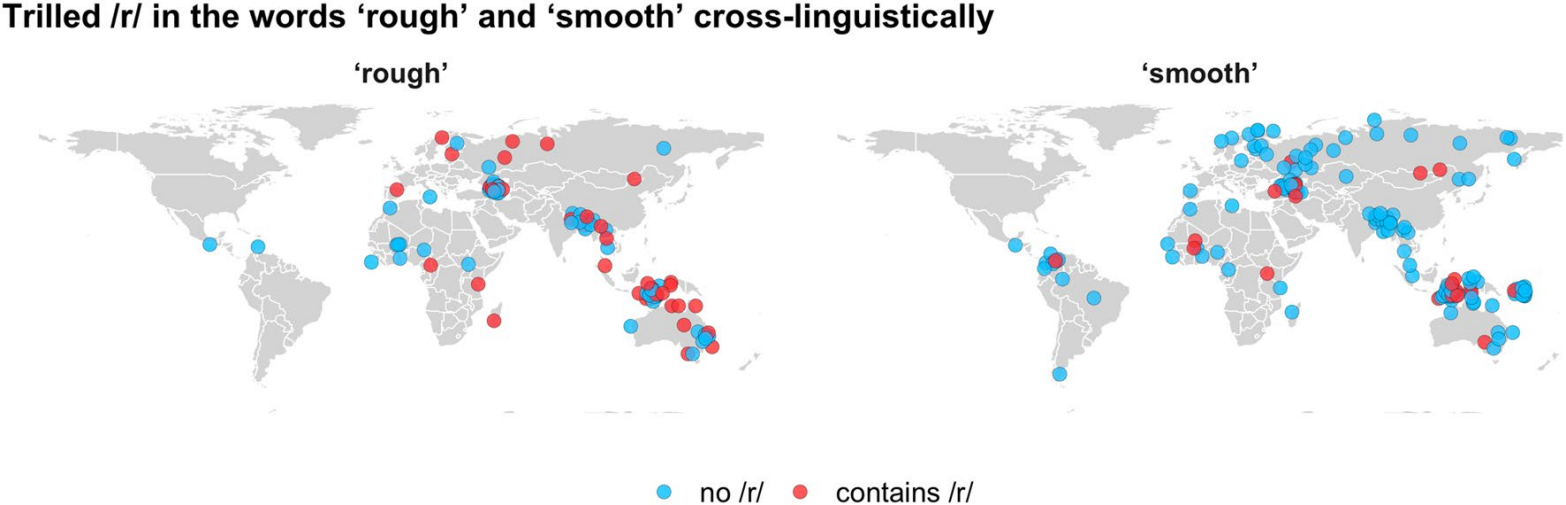
Words for the concepts ‘rough’ and ‘smooth’ in 179 spoken languages that have a trilled /r/ in their phoneme inventory, showing a much higher percentage of /r/ in rough words than smooth words.

 In Study 3, we examined whether the pattern found in English and Hungarian generalizes across a large set of the world’s spoken languages from unrelated language families. This comparison across languages also allowed us to establish whether the pattern is in fact due to trilling, or whether it occurs with any /r/, even if they are not trilled. We compiled words for the meanings ‘rough’ and ‘smooth’ from several different linguistic data bases, yielding a sample of 681 words from 332 languages representing 84 different language families (Fig. 2). For this cross-linguistic analysis, we did not include Indo-European languages, which we consider in more detail in Study 4.

**Figure 3:**
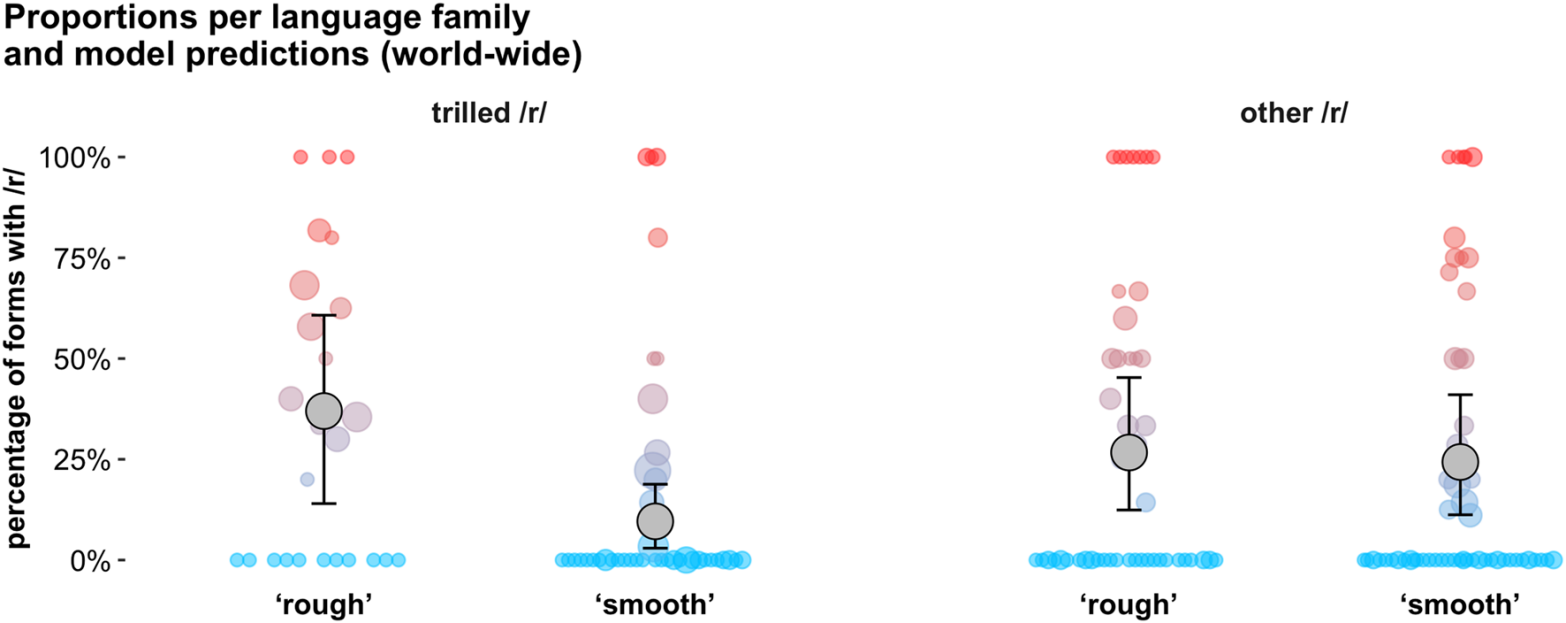
Left: Languages with only a trilled /r/ in their phoneme inventory (n = 179); Right: languages with other types of /r/ (n = 153). Results are aggregated by language family, with each colored dot representing a family (size of the circle = number of languages). Grey dots and whiskers show model predictions along with 95% Bayesian credible intervals. In languages with trilled /r/, words for ‘rough’ have a higher proportion of /r/ (posterior mean = 37%) than words for ‘smooth’ (10%); virtually no difference is observed for languages with other types of /r/.

To test our hypothesis that it is specifically trills that are associated with roughness, we compared two different types of languages with rhotics in their phonemic inventories: those with a trilled /r/, but no other rhotic sound (n = 179), and those with no trilled /r/ (n = 153) Languages without a rhotic consonant or with both trilled and non-trilled rhotics were excluded from the analysis. We fit a Bayesian mixed-effects logistic regression model to this data controlling for genealogical and areal dependencies. For languages with a trilled /r/, the estimated proportion of /r/ for the word ‘rough’ is 37% [14%, 61%], while it is only 10% [3%, 19%] for ‘smooth’, with an estimated difference of 27% [6%, 50%] (with 99.7% of posterior samples above zero; see Fig. 3). The pattern is almost completely absent for languages with a non-trilled /r/: 27% [12%, 45%] of rough words and 24% [11%, 41%] of smooth words contain a rhotic, with an estimated difference of 2% [–13%, 19%] (65.6% of posterior samples above zero). The interaction between roughness and the presence of trills is positive (1.53 in log-odds), with 98.9% of posterior samples above zero.

### Indo-European analysis

 In Study 4, we expanded our analysis to a large sample of Indo-European languages with the primary goal of evaluating the historical stability of the pattern within a single language family. This also allows us to test the assumption that the /r/ ~ rough relation in present-day English (Study 1) goes back to trilled /r/. Notably, trilled /r/ is the dominant form in many languages of this family, including Russian, Icelandic, Italian and Hindi. It is also the most likely phonetic realization of /r/ in Proto-Indo-European^56,57^.

First, to assess whether the pattern characterized the ancestral language to all modern Indo-European languages, we identified 39 reconstructed Proto-Indo-European roots related to roughness/smoothness. 44% of the roots for rough meanings contain /r/ [23%, 65%], as opposed to only 8% of the roots for smooth meanings [1%, 23%], with an estimated difference of 36% [11%, 60%] (99.75% of posterior samples over zero). This shows that the association between /r/ and roughness was already present around 6000–9500 years ago, the estimated time when Proto-Indo-European was spoken^58,59^.

**Figure 4:**
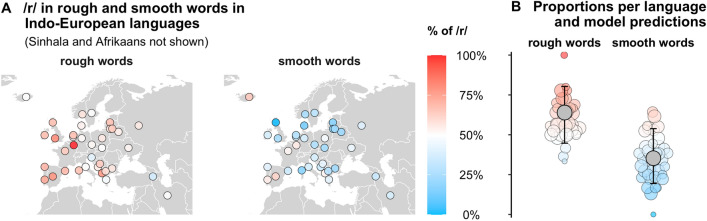
(**A**) Across the Indo-European language family, the proportion of rough words with /r/ is much higher than the proportion of smooth words with /r/; (**B**) Each dot represents a language (size of the circle = number of words); whiskers show 95% Bayesian credible intervals corresponding to the mixed-effects Bayesian logistic regression analysis indicating that rough words have a much higher proportion of /r/ (posterior mean = 63%) than smooth words (posterior mean = 35%).

Next, to assess whether the pattern is found across modern Indo-European languages, we collected quality-controlled automatic translations of the English touch adjectives of the first study from 38 Indo-European languages (including German, Spanish, Greek, Hindi, etc.), yielding a dataset of 1385 total words. In contrast to the previous analysis of Indo-European root forms, this analysis was not cognacy-controlled, but characterizes the set of words available to modern speakers of these languages. A Bayesian mixed-effects logistic regression analysis (Fig. 4) controlling for genus (Germanic, Romance, etc.) showed that /r/ is much more likely to occur in translation equivalents of rough words (63% [45%, 80%]) than smooth words (35% [19%, 54%]), with an estimated difference of 29% [7%, 48%] (99.4% of posterior samples over zero). The pattern is extremely consistent across the Indo-European family, with Icelandic being the only language in which /r/ is more frequent for smooth words. Complementing the cognacy-controlled analysis of Proto-Indo-European roots above, this analysis allows the conclusion that the over-representation of /r/ in rough words has remained stable within the Indo-European lineage for at least several thousand years.

Our hypothesis is that this evolutionary stability is due to the association between roughness and /r/ being a cultural attractor^60^, which has prevented these languages from drifting away from the seed pattern in Proto-Indo-European. An alternative possibility is that the pattern has remained stable due to inertia: the same Proto-Indo-European roots have survived in the daughter languages through inheritance and family-internal borrowing, carrying the association with them. To tease apart these two scenarios, we compared /r/ to nine other segment types that are attested across all 38 languages in our Indo-European sample of 1385 words (/l/, /m/, /n/, /s/, /p/, /b/, /t/, /d/, /k/), focusing on the variability of the association between roughness and these segments across the languages. Under the historical inertia account, we expect that any chance association (or, indeed, the lack of an association) inherited from the ancestral language will be carried over to the daughter languages with the same level of consistency as the /r/-roughness pattern. For instance, the proportion of /r/ in rough words will vary by approximately the same amount across languages as the proportion of /l/, /s/, and so on. Under the cultural attractor account, we expect that /r/ will show less variation across languages than other segments as it is anchored by its iconic association to roughness. Patterns in Proto-Indo-European lacking such an anchor will evolve in different directions in the daughter languages, leading to more variation.

We found that the proportion of /r/ in rough words varies less across languages than that of any other segment, quantified by the log-odd standard deviations across languages: /r/ = 0.69, /k/ = 0.82, /b/ = 0.83, /m/ = 0.84, /p/ = 0.91, /t/ = 0.94, /s/ = 1.10, /d/ = 1.12, /n/ = 1.23, /l/ = 1.30. To quantify cross-language variation in the strength of the /r/-roughness pattern, we first calculated the difference in log-odds of /r/ in rough vs. smooth words, and then looked at the standard deviations of this difference across languages: /r/ = 0.92, /b/ = 0.98, /t/ = 0.99, /m/ = 1.01, /d/ = 1.02, /s/ = 1.03, /k/ = 1.12, /n/ = 1.13, /p/ = 1.19, /l/ = 1.21. Together, these analyses show that the /r/-roughness pattern varies less across languages within Indo-European than other potential segmental associations with roughness. Such robust evolutionary stability is especially remarkable given that adjectives have been found to be the fastest evolving lexical class^61^.

## Discussion

Roughness is the dominant perceptual dimension of surface touch, and behavioral and neural evidence shows that people have strong cross-modal links between rough textures and sound. We examined whether the tight connection between touch and sound is reflected in the vocabularies of spoken languages, manifested in iconic correspondences between rhotic consonants and the semantic dimension of roughness. In Study 1, we found an association between /r/ and rated roughness in a large set of English sensory words, and in Study 2, we found the same pattern in a comparable set of Hungarian sensory words. Notably, Hungarian has a trilled variant of /r/, and in Study 3, we confirmed a widespread association between the trilled /r/ specifically and roughness in words for ‘rough’ and ‘smooth’ in a genealogically diverse sample of 332 spoken languages from 84 phyla. In Study 4, we looked deeper into the Indo-European family, analyzing a set of reconstructed Proto-Indo-European roots as well as sensory words of 38 modern languages. This analysis showed that the statistical association between /r/ and roughness is pervasive across the sensory lexicons of this family, and it appears to have been stable for at least 6000 years. In sum, our analyses show that the iconic /r/-roughness pattern (1) is common across unrelated spoken languages; (2) extends across sensory words within languages; and (3) can remain highly stable over time. Together these findings amount to strong evidence for a statistical universal connecting form and meaning in spoken vocabularies: the pervasive and widespread use of trilled /r/ to evoke ‘roughness’.

Our cross-linguistic evidence allowed us to isolate trilling as the critical phonetic correlate of roughness. The most parsimonious explanation for these results is that the perception of cross-modal analogies between trilling and roughness is common to people around the world and can function as a cultural attractor. This pattern is likely grounded in the acoustically and articulatorily discontinuous nature of trills, which may be associated with the intermittent discontinuity in surface texture that is known to be the primary determinant of roughness judgements^51–53^. This proposal makes testable predictions: For example, in haptic exploration tasks, listening to trilled speech sounds versus non-trilled ones should lead to higher roughness ratings, similar to how acoustic manipulations can alter perceptions of textural roughness^16,19^. And in iterated communication experiments, signals that are congruent with this cultural attractor should have a higher chance to survive^62^.

Considering that the /r/-roughness pattern is rooted in trills, English appears to be an interesting case in which the rhotic phoneme is, in most dialects, no longer trilled, and yet the language maintains a strong association between /r/ and roughness in its vocabulary. However, the iconic correspondence has not disappeared without a trace. For example, in the United States, the trilled /r/-roughness correspondence was invoked by the historic marketing slogan for the Ruffles-brand potato chip, “R-R-Ruffles have R-R-Ridges”, spoken in advertisements with an extended trilled /r/. So even without the trilled phoneme, English speakers appear to maintain a sense of correspondence between lengthened rhotic sounds and roughness, attesting to the robust underpinning of this iconic mapping.

From a historical-evolutionary perspective, our study makes several contributions to growing evidence that iconicity plays a significant role in shaping the vocabularies of languages. The prevalence of the /r/-roughness pattern across the globe, its wide distribution in lexicons, and its stability over time suggest that this iconic correspondence acts as a strong attractor in the cultural evolution of spoken languages. Previous work has shown a number of consistent form-meaning associations in word lists across spoken languages^3,4^. A critical next step is to probe the depth of such associations and the mechanisms by which they arise. Here, by combining broad analyses across diverse languages with deeper analyses of the sensory vocabularies of languages within a single family, our study sheds new light on how an iconic attractor acts to mold the vocabulary of spoken languages. Most notably, we show the potential for a strong iconic attractor to influence, not just the forms of single vocabulary items across languages, but also the forms of words across a wide domain of sensory vocabulary within a language. The finding that the association between roughness and /r/ is historically stable across thousands of years within the Indo-European language family supports the hypothesis that iconic structures are less prone to change^63^.

In conclusion, our findings highlight the perceptual domain of touch as a promising area for the quickly growing field of cross-linguistic investigations of iconicity^34–36^, building on language-specific descriptions of particular patterns^33,34^ and work on more coarse-grained phonological and semantic domains^4,64^. More generally, our results contribute to growing evidence of deep connections between audition and touch, which are attested behaviorally^16^, neurally^22^, and linguistically^30,31^. The evidence presented here shows that /r/-for-rough is one of the most robust, cross-linguistically consistent, and evolutionarily stable patterns of iconicity discovered to date: a new statistical universal of spoken language linking speech and touch, and shaping the very texture of the lexicon.

## Materials and methods

### Statistical analysis software

We use the R statistical programming environment^65^ version 4.0.2 and the tidyverse package^66^ version 1.3.0 for data processing and statistical analysis. Random forests were implemented with the ranger package^67^ version 0.12.1. Bayesian multilevel regressions were implemented using brms^68^ version 2.13.3.

### English analysis

Our initial analysis of English relied on an existing set of 100 English adjectives from a rating study conducted by Stadtlander and Murdoch^13^. The word *wafflish* was excluded from analysis because it is not listed as a word in most dictionaries, including the Oxford English Dictionary. The word list was constructed by asking native speakers of American English to list as many adjectives as possible that can be used to describe perceptual properties (such as roughness, hardness, size, etc.). Out of the 440 total words that were obtained this way, 100 adjectives were then rated for roughness by a separate set of participants.

The meaning of words was rated on a scale from − 7 (complete lack of roughness = smooth) to + 7 (maximum roughness) by 20 speakers. The average correlation between speakers is high (*r* = 0.7). We paired the average roughness rating of words with phonemic transcriptions taken from the English Lexicon Project^69^ (transcriptions were manually checked by the first author). The transcriptions were used to create indicator variables coding for the presence/absence of each phoneme within each word (as most words in our data set are short, sounds are rarely repeated, and therefore tallying the exact number of occurrences of the same phoneme within a word does not yield a superior predictor over and above a dichotomous variable). The indicator variables obtained this way were all used as predictors in a random forest^41^, with roughness rating as the dependent measure. We chose to analyze our data using random forests (rather than, for example, a regression model) in order to deal with the ‘low *n*, high *p*’ nature of our data (many different predictor variables, few words), where random forests have been argued to outperform standard regression models^42^. Random forests are furthermore preferred due to potential collinearities in the data, as phonemes often tend to occur together in particular combinations. This analysis used an ensemble of 5000 trees. We calculated relative variable importance using permutation. Our analysis considered all phonemes within the entire word.

To statistically evaluate the relationship between the presence of /r/ and roughness, we fitted a Bayesian logistic regression model to the data with presence of /r/ as the outcome and roughness as the only predictor variable. For the purposes of this model, roughness was coded as a binary predictor with the values ‘rough’ for words with roughness ratings higher than or equal to 0 and ‘smooth’ for words with roughness ratings lower than 0. Dichotomizing the variable has a two-fold motivation: first, the roughness ratings are *highly* bimodal, with a near perfect split between words that are dominantly smooth and words that are dominantly rough. Second, our other analyses (see below) use a similar binary classification because when analyzing rough/smooth meanings across languages, we cannot assume that the precise roughness values and their relative rankings (such as whether *rocky* is rougher than *rugged*) carry over to other languages. Moreover, in the worldwide analysis, we only have meanings for ‘rough’ and ‘smooth’, the endpoints of the continuum. Thus, to use the same analytic approach throughout the paper, we opted to dichotomize the roughness predictor. Importantly, the same results are obtained when the variable is not dichotomized (continuous predictor). In our Bayesian logistic model, we used weakly informative priors for the intercept and the binary roughness predictor: student’s *t* distributions with the degrees of freedom parameter set to 5, the location parameter to 0, and the scale parameter to 2.5.

We additionally compared the relative proportion of /r/ in adjectives for rough surfaces to (1) a baseline of general English adjectives, and (2) a widely used data set of 423 sensory adjectives^43^. For the general English adjectives, we used all the words from the Carnegie Mellon University dictionary^70^ which we filtered based on corpus-derived part-of-speech tags^71^ for words that were used as adjectives 80% of the time. This left 5806 words altogether. Of our set of sensory adjectives, we were able to obtain phonemic transcriptions for 386. We computed the proportion of /r/ for these two datasets to act as a comparison to the rough words we consider.

### Hungarian analysis

The aim of the Hungarian analysis was to collect roughness ratings for a different language that was not related to English. For the sake of making the results comparable, the design is largely informed by Stadtlander and Murdoch’s original study^13^. The second author of this paper (a native speaker of Hungarian) first created a list of 85 Hungarian adjectives that describe surface properties. The majority of these words were obtained by translating the adjectives on Stadtlander and Murdoch’s list into Hungarian automatically using Google Translate. The translations were manually checked and edited by the second author. A few additional missing surface descriptors were then added by (1) searching the Hungarian National Corpus^72^ for words followed by the word *felszín* ‘surface’; (2) manually identifying actual surface descriptors in the resulting list; and (3) merging this list with the translations. Hungarian phonemic transcriptions were added manually by the second author.

The experiment was approved by the Ethics Committee of the Department of Language and Linguistic Science at the University of York (where this experiment was originally conducted). All methods were carried out in accordance with the Code of Practice and Principles for Good Ethical Governance of the University of York. All participants provided informed consent before participating in the experiment. Participants were 21 native speakers of Hungarian, recruited from a university course at Eötvös Loránd University (Budapest; all over the age of 18). The study was administered using Qualtrics. Participants were instructed to rate each word on a scale of − 7 to + 7, with − 7 indicating a completely smooth surface, and + 7 indicating a very rough surface. They were also instructed to try to imagine what it would feel like to touch the surface and base their answers on their intuitive impression.

One participant was excluded from the analysis after an interrater reliability analysis found their ratings to be negatively correlated with those of other participants (indicating that they failed to follow the task instructions). All other participants were strongly correlated with each other (the mean correlation coefficient is *r* = 0.68 ranging between 0.40 and 0.86). We note that our analysis yields the same results even if this participant is kept in the data set.

The random forest specification was the same as in the English analysis, except that we excluded those words that are known to be borrowed from Indo-European based on an etymological dictionary of Hungarian^73^ (a random forest analysis including the Indo-European words also shows /r/ to be highly predictive of roughness). The logistic regression model for testing the relationship between /r/ and roughness was specified in exactly the same way as in the English analysis.

### Cross-linguistic analysis

The full data set for the cross-linguistic analysis comes from four different sources: Google Translate (processed in the way described above), CLICS^74^, Chirila^75^ and RefLex^76^. All of these data sets contain (1) word forms and (2) their translations and/or definitions in English (and/or French in the case of RefLex). Relevant words were extracted using a variety of different strategies depending on the data.

- Google Translate: As explained above, these data were generated by translating the words from the Stadtlander and Murdoch^13^ list into the target language, and then checking whether they successfully back-translated into English. Therefore, these data contain a subset of the Stadtlander and Murdoch adjectives for each language. For the global analysis, only non-Indo-European languages were used.
- RefLex: This data set provides definitions of words in English/French. For English, we extracted all definitions that contained any of the words from Stadtlander and Murdoch. For French, all definitions with the words *rugueux* ‘rough’ and *lisse* ‘smooth’ were extracted. Lexical data was downloaded from RefLex with an eye for (1) lexicon size and (2) phylogenetic diversity. We first selected sources with > 5000 records in RefLex; there were 36 of these in July 2017. Within that sample, we prioritized sources according to linguistic diversity, striving to select representatives from the major African phyla and subphyla recognized by Glottolog. When there are multiple sourcs for one languoid or language cluster (e.g., the closely related Wolof, Sereer, Fulfulde) we prioritized the source with the biggest number of records and exclude the others to avoid duplicate or near-duplicate lemmas. When there are sources for multiple languages in a larger, more internally diverse group (e.g., Bantu, Dogon), we selected multiple based on number of records and group-internal diversity.

Due to our search methods, this is a noisy data set, as not all words whose definition contains our target words are *bona fide* surface descriptors. We therefore (1) manually checked the entire list and only retained words where the definition made it clear that they unambiguously referred to a surface descriptor; (2) replaced the definitions with translations based on the definitions; and (3) eventually limited this data set to words that translate as ‘rough’ and ‘smooth’. The scripts presented in the OSF repository (https://osf.io/6nma2/) contain a detailed track record of all individual classifications we undertook.

- Chirila: This data set provides translations of words in English. We obtained permission to use the Chirila data from the creator of this data base, Prof. Claire Bowern. The data was shared with us on August 25, 2017. We extracted all words whose translations contained any of the words from Stadtlander and Murdoch^13^. This, again, yielded a somewhat noisy data set that includes a number of irrelevant items (e.g., verbs). Our analysis only used those words whose translation is simply ‘rough’ or ‘smooth’ without any additional text.
- CLICS (version 3): CLICS is a database of cross-linguistic co-lexifications which provides translations of words in English. The database was originally compiled for the purpose of studying co-lexification patterns across the languages of the world, and is available in a standardized form following the Cross-Linguistic Data Formats (CLDF) initiative^77^. Note that CLICS itself consists of 30 different preexisting data sets. In order to maximize the reproducibility and transparency of our analyses, we did not manually download the data via the CLICS website, but instead accessed the 30 component data sets using a Python script (clics_cldf_extract_data.py in the data processing folder of the “Supplementary materials S1”). 29 of the 30 data sets were accessed through the Lexibank database using v0.2 of the lexibank-analysed GitHub repository (https://github.com/lexibank/lexibank-analysed^78^). The remaining one data set (LexiRumah v3.0.0;^79^) was also accessed using CLDF tools, but through a separate repository (https://github.com/lessersunda/lexirumah-data) as it was not yet part of the Lexibank database at the time of analysis. We extracted all words that translate either as ‘rough’ or ‘smooth’, and corresponding phonemic or orthographic transcriptions as they were represented in the data sets.

As mentioned above, we limited the large-scale cross-linguistic analysis to words that translate directly as ‘rough’ and ‘smooth’, because (1) for a large proportion of languages, only these two words were available in our data; and (2) both of these words are completely unambiguous in terms of their roughness rating (as opposed to e.g., words like the English *blunt*, *woolly*, and *textured*, which had highly ambiguous roughness ratings close to 0). A small minority of languages had multiple words for ‘rough’ and/or ‘smooth’, which are all included in our analysis (for the purposes of Fig. 3, these languages were collapsed to a single data point, following a majority rule: if the majority of the words had an /r/, the data point is shown in red, otherwise it is shown in blue).

For each language, we obtained information about language family, linguistic area, latitude and longitude from the AUTOTYP cross-linguistic database^80^. In addition, each language was coded for rhotic-type using a combination of information from the PHOIBLE phonological inventory database^81^ and our own phonetic judgments based on existing reports and reference grammars for each language. In establishing the type of rhotic, we took a conservative approach. Languages with a single trilled rhotic or multiple contrastive trilled rhotics were coded as “trilled /r/”; languages with at least one non-trilled rhotic were coded as “other” (this includes languages that have both trilled and non-trilled contrastive rhotics); and languages without rhotics were coded as “no /r/”. In our analysis, we contrast the first two types of languages: those with a trilled /r/ only and those with other types of /r/.

To summarize, our data set included words that translate as ‘rough’ or ‘smooth’. Only words from languages with at least one rhotic phoneme were included. We further limited this data set to non-Indo-European languages only, as Indo-European languages were considered separately in another analysis (see below). After these exclusions, our final data set contained 681 words from 332 languages (179 with trills only and 153 with other rhotic types) representing 84 language families and 19 AUTOTYP areas. We note that there were very few languages from North America in our final sample (especially in the trilled /r/ group, cf. Figure 3). This is not because these languages are not present in the source data sets, but because few of them have rhotics, and even fewer trills.

We fitted a Bayesian mixed effects logistic regression model to these data with presence of /r/ as the outcome. The fixed effect predictors were (1) whether the language had trilled /r/ or not; (2) a binary roughness variable; and (3) their interaction. The model also included random intercepts by language family and linguistic area, and random slopes over trilled /r/, roughness, and their interaction by family and area. We used the same priors as in the analysis of the Indo-European data.

### Indo-European analysis

We used Google Translate to generate automatic translations of the 99 English adjectives from Stadtlander and Murdoch^13^ into all languages represented on Google Translate. For the current analysis, we further limited the data set to Indo-European languages (the non-Indo-European languages on Google Translate were included in the cross-linguistic analysis described in the prior section). To make sure that there is a sufficient degree of translational equivalence, we only included words that successfully translated from English into the target language and back into English. For example, *velvety* was translated into German as *samtig* and using this German word as the basis for a translation into English yields *velvety* again. However, the English word *ragged* translated into German as *zottig*, which, when translated back into English, yielded a different word: *shaggy*. Using only those words that successfully back-translated also helps to ensure that we focus on the correct meaning for polysemous English words in our cross-linguistic analysis. It should be noted that, as a consequence of this conservative approach, our analysis presumably under-represents the extent of different languages’ touch vocabularies as we can only capture those words that are represented in English. We removed languages where fewer than ten words successfully back-translated into English (the low rate of successful back-translations is an indicator of poor overall translation quality). For each language, we also added information about genus within the Indo-European family (Germanic, Romance, Slavic etc.) based on the AUTOTYP cross-linguistic database^80^.

To ensure that our automated translations are of sufficiently high quality, we enlisted native speakers to manually check the translations for five out of the 38 Indo-European languages in the data set (German, Dutch, Spanish, Polish and Italian). The native speakers were the authors themselves for German and Dutch, and other linguists for Spanish, Polish and Italian. We used three labels: “good” for perfect translations that would also have been the native speakers’ first choice; “ok” for acceptable translations that (1) include the right stem but with minor issues in the suffixes or (2) are appropriate as a translation but would not have been the native speakers’ first choice; and “bad” for translations that are clearly wrong. Averaging across the five languages, 93% of the translations were judged as “ok” or “good”, and 77% as “good” (i.e., perfect translations). When only “good” translations are considered, the proportions of words with /r/ in the rough vs. smooth groups shift only by 3% per language on average. In fact, when only “good” translations are kept, the strength of the main pattern increases for this subset of five languages: in the original data, the difference in the proportion of /r/ in rough vs. smooth words is 13.5% (for these five languages); when only perfect translations are considered, the same difference is 15%.

The data was analyzed using a Bayesian mixed effects logistic regression model with presence of /r/ (established using regular expressions based on orthographic forms tailored to each language) as the dependent variable and roughness (coded as a binary variable as before, based on the original ratings from English) as the only fixed effect predictor variable. We included random intercepts by genus, language and word, as well as random slopes over roughness by genus and language. The same priors were used for the fixed effects as before; we used an LKJ prior for the random effects correlation matrix with *eta* = 2; and the prior for the random effect standard deviations was a half-Student’s *t*-distribution with degrees of freedom = 4, location = 0 and scale = 2.

To test whether the same pattern was present in Proto-Indo-European, we performed an additional analysis of Proto-Indo-European roots in the Penn Linguistics/Price Lab Etymological Database System (PLEDS, Rolf Noyer)^82^. We thank Rolf Noyer for giving us access to this data. We limited the set of forms in the database to adjectives only, and used regular expressions to extract all forms whose meanings contained any of the English surface descriptors from Stadtlander and Murdoch^13^. We categorized these forms as ‘rough’ and ‘smooth’ based on the ratings in Stadtlander and Murdoch. Only unique roots were included in our final data set. We analyzed the data using a Bayesian logistic regression model with the presence/absence of /r/ in the reconstructed roots as the dependent variable and roughness as the only predictor variable. The same priors were used as in our models for English and Hungarian described above.

In addition, we used regular expressions to create binary variables indicating the presence/absence of the following nine segment types in each word in the Indo-European translations database: /l/, /m/, /n/, /s/, /p/, /b/, /t/, /d/, /k/. Since many of the languages in our sample have complex mappings between orthography and phonological forms, special care was taken to design regular expressions that only capture the target segment. For instance, /k/ in French was matched via the following regular expression: (k|c$|c[^ie]|q). This captures orthographic <k> in any position, <c> at the end of the word or when not followed by <i> or <e> and <q>. We also note that a given “segment type” may actually correspond to more than one phonetic realization. Thus, /t/ encompasses both the aspirated /t^h^/ found in Icelandic, Swedish and English, and the unaspirated /t/ found in Dutch, French and Spanish; and /d/ encompasses both the unaspirated voiceless stop /t/ in Icelandic, and the unaspirated voiced stop /d/ in Dutch, French, etc. While these sounds may not be phonetically equivalent, our justification for grouping them together is that they play very similar roles in the phonological systems of the languages we consider. This is in line with the strategy used for /r/, where phonetically different segments (e.g., uvular fricatives in French and the alveolar trill in Spanish) are grouped together based on their phonological behavior.

Using the binary indicator variables above, we calculated the proportion of each segment in rough versus smooth words in each language. Since our goal was to estimate how much these proportions vary across languages, and since proportions that are close to the boundary values of 0 and 1 necessarily show less variation than proportions in the middle of the continuum (e.g., 0.5), we transformed all proportions to log-odds. This helps to avoid artificial boundary effects. Since proportions of 0 and 1 cannot straightforwardly be represented using log-odds, but they are observed in some cells in our data, we first replaced such proportions with 0.03 and 1 − 0.03 = 0.97. The value of 0.03 (i.e., 3%) was chosen as this is the whole-valued percentage that is the closest to the second lowest proportion observed in our data. Using different small values yields minor quantitative differences but qualitatively the same results.

Using the by-language log-odds, we first calculated the standard variation of the log-odds of each segment type across languages. We then calculated the standard deviation of the difference in the log-odds of each segment between rough versus smooth words (i.e., a measure of the size of the roughness effect for each segment in each language).

## Data Availability

All data, code, and materials used in the analysis are openly available in the OSF repository at https://osf.io/6nma2/.
